# Kinematic Monitoring of the Thorax During the Respiratory Cycle Using a Biopolymer-Based Strain Sensor: A Chitosan–Glycerol–Graphite Composite

**DOI:** 10.3390/bios15080523

**Published:** 2025-08-09

**Authors:** María Claudia Rivas Ebner, Emmanuel Ackah, Seong-Wan Kim, Young-Seek Seok, Seung Ho Choi

**Affiliations:** 1 Department of Biomedical Engineering, Yonsei University, Wonju 26493, Republic of Korea; crivasebner@yonsei.ac.kr (M.C.R.E.); eackah.3@yonsei.ac.kr (E.A.); 2 Department of Agricultural Biology, National Institute of Agricultural Sciences, Rural Development Administration, Wanju 55365, Republic of Korea; tarupa@korea.kr; 3 Gangwon-do Agricultural Product Registered Seed Station, Chuncheon 24410, Republic of Korea; 4 Department of Integrative Medicine, Major in Digital Healthcare, Yonsei University College of Medicine, Seoul 06229, Republic of Korea

**Keywords:** strain sensor, wearable device, respiratory monitoring, chitosan-graphite, health monitoring

## Abstract

This study presents the development and the mechanical and clinical characterization of a flexible biodegradable chitosan–glycerol–graphite composite strain sensor for real-time respiratory monitoring, where the main material, chitosan, is derived and extracted from *Tenebrio Molitor* larvae shells. Chitosan was extracted using a sustainable, low-impact protocol and processed into a stretchable and flexible film through glycerol plasticization and graphite integration, forming a conductive biocomposite. The sensor, fabricated in a straight-line geometry to ensure uniform strain distribution and signal stability, was evaluated for its mechanical and electrical performance under cyclic loading. Results demonstrate linearity, repeatability, and responsiveness to strain variations in the stain sensor during mechanical characterization and performance, ranging from 1 to 15%, with minimal hysteresis and fast recovery times. The device reliably captured respiratory cycles during normal breathing across three different areas of measurement: the sternum, lower ribs, and diaphragm. The strain sensor also identified distinct breathing patterns, including eupnea, tachypnea, bradypnea, apnea, and Kussmaul respiration, showing the capability to sense respiratory cycles during pathological situations. Compared to conventional monitoring systems, the sensor offers superior skin conformity, better adhesion, comfort, and improved signal quality without the need for invasive procedures or complex instrumentation. Its low-cost, biocompatible design holds strong potential for wearable healthcare applications, particularly in continuous respiratory tracking, sleep disorder diagnostics, and home-based patient monitoring. Future work will focus on wireless integration, environmental durability, and clinical validation.

## 1. Introduction

Chitosan, a linear polysaccharide composed of β-(1→4)-linked D-glucosamine and N-acetyl-D-glucosamine units, is derived via the deacetylation of chitin—a structural polymer abundant in crustacean exoskeletons, fungal cell walls, and insect cuticles [1,2]. While crustacean sources dominate industrial production, insects such as *Tenebrio Molitor* larvae have emerged as a sustainable alternative, offering distinct advantages such as lower mineral content (reducing demineralization steps) and scalable farming with minimal environmental impact [2,3]. The degree of deacetylation (DD) during processing critically determines chitosan’s solubility, mechanical strength, and bioactivity, enabling tailored properties for biomedical applications [1,3]. In the medical field, chitosan’s biocompatibility, biodegradability, and cationic charge underpin its use in wound dressings, drug delivery systems, and tissue engineering scaffolds [4,5]. These properties arise from its amino and hydroxyl groups, which facilitate electrostatic interactions with mammalian cells, enhancing muco-adhesion and promoting regenerative processes [4,5]. Recent innovations in soft electronics further exploit chitosan’s film-forming ability, compatibility with plasticizers like glycerol, and capacity for conductive filler integration, positioning it as a versatile substrate for wearable sensors [6,7].

The shift toward insect-derived chitosan aligns with circular economy principles [4,8]. *Tenebrio molitor* exoskeletons contain 10–15% chitin by dry weight, requiring 30% fewer chemicals during extraction compared to crustacean sources [2,4]. This streamlined process yields medical-grade chitosan with comparable purity and bioactivity, while insect farming’s low carbon footprint addresses sustainability challenges in biomaterial production [4,9]. To adapt chitosan for use in stretchable sensor design, its mechanical flexibility is enhanced by incorporating glycerol, which disrupts hydrogen bonding between polymer chains and increases elasticity [9,10]. The addition of graphite introduces electrical conductivity through percolation networks, enabling the composite to transduce mechanical deformation into quantifiable electrical signals [10,11]. The sensitivity of these chitosan–glycerol–graphite composites is governed by the dispersion of fillers and interactions at the matrix–filler interface [7,11]. Stretch sensors have become indispensable in medical diagnostics and patient monitoring, particularly for tracking physiological movements that reflect underlying pathologies [12,13]. For instance, in respiratory care, they enable non-invasive detection of thoracic expansion patterns associated with obstructive or restrictive lung diseases, for example [12,14].

**Figure 1:**
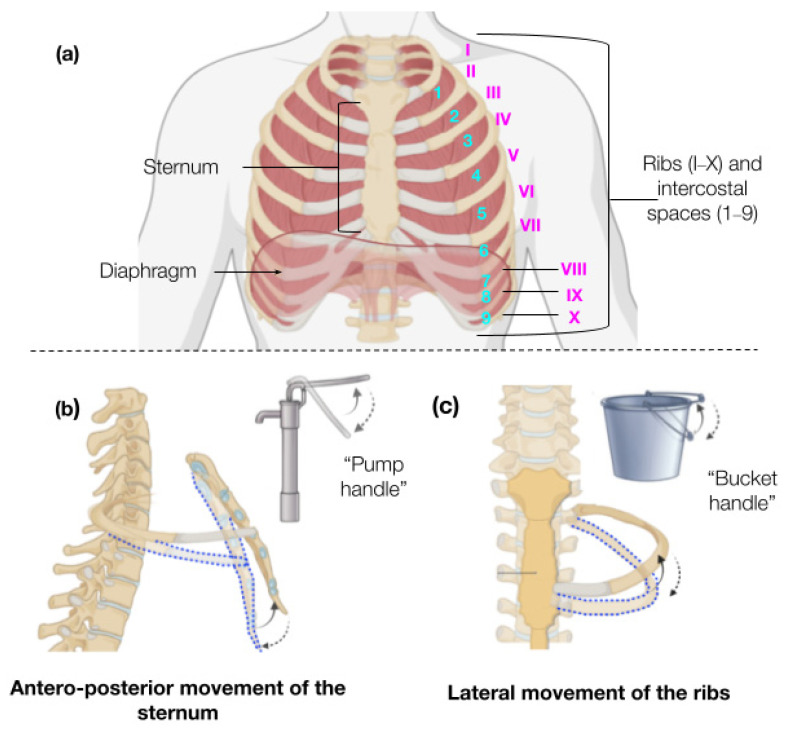
(**a**) The basic musculoskeletal structures of the thorax, including the sternum, ribs, intercostal muscles, and diaphragm, are depicted. Intercostal spaces are highlighted in blue, and ribs are labeled using Roman numerals (I–X). (**b**) The mechanical movements of the thoracic cage are illustrated, showing the ‘pump handle’ motion of the sternum and the (**c**) ‘bucket handle’ motion of the lower ribs during respiration.

To understand different monitoring techniques, basic comprehension of thoracic biomechanics and respiratory physiology is needed. In Figure 1, we can see that the thorax is formed by the thoracic vertebrae, the ribs (24 ribs in total, 12 in each hemithorax), and the sternum (see attached image), with functions including protection of vital internal organs, muscle attachment, and its major role in ventilation [15]. These structures work in coordinated motion to produce respiration. Respiration is a complex process where the respiratory system (mouth, nose, pharynx, larynx, trachea, bronchi, bronchioles, and the functional unit, the alveoli) and the thoracic wall interact, enabling lung expansion [16]. During each expansion (inhalation) and relaxation (passive exhalation), gas exchange occurs (inhalation of O_2_ and exhalation of CO_2_) between the alveoli and blood capillaries (see gas exchange image) [16]. The constant lung expansion during breathing results from thoracic wall movement and respiratory muscles, primarily the diaphragm and intercostal muscles, which increase negative pressure in the thoracic cavity, drawing air in [17]. A respiratory cycle—defined as one complete sequence of inspiration (inhalation) and expiration (exhalation)—involves biomechanical interactions between the thoracic musculoskeletal system, producing two main movements: the “pump handle” movement of the superior ribs (1–4) and sternum, increasing the transverse chest diameter, and the “bucket handle” movement of the lower ribs, increasing the anteroposterior chest diameter. Figure 1b,c illustrate this mechanism [18,19]. During inhalation, these movements expand the thorax, while during passive exhalation (typically muscle-independent in healthy individuals), the thorax returns to its resting position [20]. These dynamics drive pressure and volume changes in the lungs. The tidal volume—the air moved during a normal breath—is approximately 500 mL for men and 400 mL for women, with a healthy respiratory rate of 12–20 cycles per minute [15]. Pathologies can alter tidal volume or respiratory rate (e.g., tachypnea, hyperpnea) [20]. Current clinical monitoring methods focus on detecting volume (L), flow (L/min), and pressure (cmH_2_O) changes during respiratory cycles (common in invasive mechanical ventilation), capnography (exhaled CO_2_ sensing), or non-invasive methods like ECG, an indirect measurement of the respiratory rate, and suffer from 40 to 60% error rates due to motion artifacts and poor adhesion, often failing to detect critical events like the Kussmaul respiratory pattern [21,22,23,24]. In contrast, chitosan-based stretch sensors conform to skin topography, directly correlating resistance changes with chest wall displacement [6,9]. A 1% strain in the sensor generates measurable resistance shifts, enabling precise tracking of the tidal volume and respiratory rate [9,11]. To address the material’s nature, a straight-line sensor geometry was adopted. This design minimizes stress concentration points that arise in complex patterns (e.g., kirigami), thereby reducing the risk of crack propagation during repetitive stretching. The linear configuration ensures uniform strain distribution across the sensor, preserving electrical continuity even under cyclic loading (up to 500 cycles in preliminary testing). The glycerol-plasticized chitosan matrix exhibited brittleness under high deformation, making simplified geometries more suitable for maintaining structural integrity [25,26,27,28]. The straight-line design prioritizes reliable signal transduction over extreme stretchability, aligning with the sensor’s intended application in low-strain respiratory monitoring (1–5% thoracic expansion). Also, strain during deep breathing and coughing has been reported to range from 4 to 8% in normal respiration and up to 12–16% in pathological or forced respiratory maneuvers. Other sensors with similar components as that in this study have demonstrated documented sensor stability over 10,000 cycles at 15% strain with less than 5% signal degradation, and graphite composite strain sensors have confirmed consistent performance over 20,000 respiratory cycles simulated at 10% and 15% strain. Considering this, for optimal thoracic expansion sensing, sensors were positioned at the sternum (4th intercostal space) and lower ribs (6th–9th intercostal spaces) to capture anterior and anterolateral thoracic movements [25,28]. As confirmed in recent reviews, wearable and sustainable biopolymer-based sensors offer superior skin conformity, real-time physiological signal tracking, and improved long-term user comfort compared to conventional synthetic systems. Stretch sensors have become indispensable in medical diagnostics and patient monitoring, particularly for tracking physiological movements that reflect underlying pathologies. For instance, in respiratory care, they enable non-invasive detection of thoracic expansion patterns associated with obstructive or restrictive lung diseases [29,30,31,32,33]. Chitosan’s unique molecular structure, with abundant amino and hydroxyl groups, supports strong interactions with plasticizers such as glycerol and conductive fillers like graphite, carbon nanotubes, or metallic nanowires. This chemical versatility allows the engineering of flexible, breathable films—via solution casting, extrusion, or electrospinning—that form the basis for on-skin wearable electronics. Incorporation of glycerol disrupts hydrogen bonding and enhances mechanical flexibility, while the percolation networks formed by conductive fillers confer tunable conductivity, enabling strain sensing, electrophysiological recording, and soft actuation. The optimization of filler dispersion and chitosan molecular weight, as shown in recent studies, results in sensors that provide a high gauge factor and robust fatigue resistance—both critical for long-term healthcare and sports applications. Nevertheless, significant limitations remain in current respiratory monitoring: existing technologies are often invasive or, when non-invasive (such as ECG-derived respiratory monitoring), suffer from inaccuracy, poor adhesion, or impractical design. Many of these systems are also expensive and require professional operation, limiting their accessibility and practicality. Thus, this study aims to fill this gap by developing a comfortable, cost-effective, and user-friendly chitosan-based sensor with improved patient adhesion for reliable, non-invasive respiratory cycle monitoring [4,7,9].

## 2. Materials and Methods

### 2.1. Materials

All materials used in this study were carefully selected to ensure the reproducibility and quality of the experimental procedures. Mealworm (*Tenebrio Molitor*) shells, serving as the primary biopolymer source, were obtained from Gangwon-do Agricultural Product Registered Seed Station, Chuncheon, Republic of Korea. For chemical processing and composite fabrication, extra pure sodium hydroxide (NaOH, OCI Company Ltd., Seoul, Republic of Korea), glacial acetic acid (CH_3_COOH, Daejung Chemicals & Metals Co., Ltd., Siheung, Republic of Korea), and glycerol (C_3_H_8_O_3_, ≥99.5%, Sigma-Aldrich, St. Louis, MO, USA) were employed. Synthetic graphite powder (<20 µm, Sigma-Aldrich) was used as the conductive component. All reagents were of analytical grade and used as received without further purification.

### 2.2. Extraction of Chitosan

**Figure 2:**
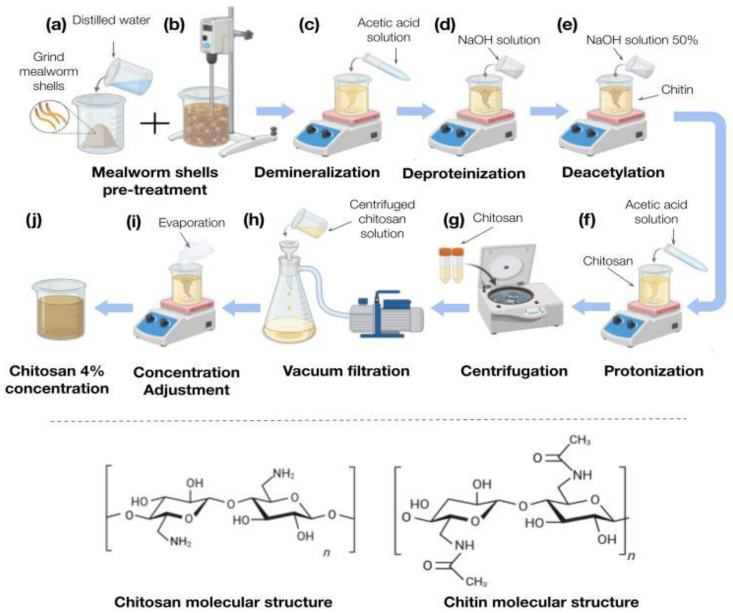
Chitosan extraction process from *Tenebrio molitor* shells. (**a**) Raw mealworm shells are used as starting material. (**b**) Shells are ground, hydrated, and stirred. (**c**) Acetic acid is applied to initiate demineralization. (**d**) Deproteinization is performed to remove residual proteins. (**e**,**f**) The material undergoes further treatment with acetic acid and NaOH to purify and deacetylate chitin. (**g**) The solution is centrifuged to separate insoluble components. (**h**) Filtration is carried out using a vacuum pump. (**i**) The extract is concentrated by evaporation to (**j**) yield a 4% chitosan solution.

Our main novel contribution in this project is the use of the biopolymer called chitosan. To extract it from the shell of the *Tenebrio Molitor* larvae, a step-by-step process must be followed. As Figure 2 shows, the process was divided into 7 steps to make it simpler: new sample preparation, demineralization, deproteinization, deacetylation, final demineralization, filtering process, and adjustment of concentration.

#### 2.2.1. New Sample Preparation

Mealworm shells (25 g) were weighed, grinded, and transferred to an 1800 mL beaker. The shells were ground into powder using an electrical hand blender (PHILIPS Hand Blender 3000 series, HR2520/00, Amsterdam, The Netherlands), particle size was not measured but visually confirmed to be coarse fine powder. The fine powder was cleaned with distilled water (750 mL) during 12 h at 70 °C, with continuous electrical stirring. After these 12 h heating process, the hydrated shells were separated from the distilled water by filtration through a standard laboratory strainer. The filtered shells were cleaned again with cold distilled water, stirred manually, and filtered again. This washing process was performed 5 times before starting the next step. In this protocol, each step (except filtering and concentration adjustment steps) is followed by this washing process. The “new sample preparation” step is illustrated in Figure 2a,b).

#### 2.2.2. Demineralization

Figure 2c shows the demineralization step, which was performed via acid treatment using acetic acid. This step removes mineral components—primarily calcium carbonate—from the exoskeleton to isolate chitin and chitosan. The reaction between acetic acid and calcium carbonate produced calcium acetate, water, and carbon dioxide, thereby eliminating minerals from the mealworm shell waste. Demineralization is critical for ensuring the purity of extracted chitin, as residual minerals can compromise material quality. To minimize depolymerization of chitin and chitosan, a specific reaction duration and temperature were chosen. To remove mineral content (mainly calcium carbonate), 35 mL of glacial acetic acid (≥99.7%, Daejung Chemicals & Metals Co., Ltd., Siheung, Republic of Korea) was mixed with 565 mL of distilled water to yield a 5.8% *v*/*v* acetic acid solution (final volume: 600 mL). The hydrated shells were transferred into a 1000 mL beaker, and the acetic acid solution was slowly added over them. The suspension was covered and stirred magnetically at 70 °C for 12 h. After treatment, the solids were filtered and washed five times with distilled water as described in Section 2.2.1.

#### 2.2.3. Deproteinization

Deproteinization methods for insects involve removing proteins and organic matter to isolate chitin and chitosan. These present protein molecules are linked to the chitin structure via glycosidic bonds, and sodium hydroxide breaks them. Studies have used varying concentrations of NaOH, typically ranging from 1 M to 4 M, and high temperatures (70–100 °C) are commonly used for deproteinization. This helps improve this process efficiency, especially for more resistant proteins. In total, 50 g of sodium hydroxide (NaOH) pellets were slowly dissolved in 150 mL distilled water with continuous stirring until fully dissolved. This concentrated NaOH solution was poured into a beaker containing the washed, semi-wet *Tenebrio molitor* larval shells (who occupied 150–200 mL of volume); then, distilled water was added until the total volume reached 500 mL. Therefore, the effective NaOH concentration during deproteinization was approximately 3.6–4.2 M. The mixture was stirred at 300–400 rpm, covered, and incubated at 70 °C for 24 h. After reaction, the residue was filtered and washed repeatedly until reaching a neutral pH.

#### 2.2.4. Deacetylation

Deacetylation converts chitin into chitosan. The process involves the removal of the acetyl groups attached to the amino group to expose the -NH_2_ groups and results in a polymer containing both *N*-acetyl-glucosamine units and glucosamine. This step is critical, because it not only affects acid–base behavior, electrostatic characteristics, biodegradability, self-aggregation, solubility, sorption properties, and the ability to eliminate metal ions, among many other properties, but also determines its classification and affects its suitability for specific applications. Deacetylation methods for insects involve the removal of acetyl groups from chitin to produce chitosan. NaOH is the most used solvent for deacetylation.

The chitin obtained after deproteinization was subjected to alkali deacetylation by suspending it in 200 mL of 50% (*w*/*v*) NaOH solution prepared by dissolving 100 g NaOH pellets in distilled water. This concentrated alkali solution was poured over the chitin placed in a 500 mL beaker, bringing the total volume to approximately 300 mL. The mixture was stirred continuously at 300–400 rpm and heated at 120 °C for 24 h in a covered vessel to minimize evaporation. Following the reaction, the chitosan precipitate was filtered using a paper filter and washed thoroughly five times with distilled water until the washings reached a neutral pH, ensuring the removal of residual alkali.

#### 2.2.5. Protonation

Chitosan is made by partly deacetylating chitin. Chitosan exhibits insolubility in both water and solvents made from organic matter; however, it readily dissolves in acidic solutions with a pH lower than 6.5. At an acidic pH (in this case, with acetic acid), chitosan is protonated (protonation of the glucosamine amino unit (-NH_2_), which converts into glucosamine-NH_3_^+^), and its solubility increases due to electrostatic repulsion between the protonated amino groups. A higher concentration of acetic acid may promote the solubilizing of chitosan into the solution, which may lead to smoother film formation. A higher acid concentration leads to a higher degree of protonation. The mealworm-derived chitosan product was mixed with a 4% (*v*/*v*) acetic acid solution to solubilize and prepare it for further analysis or applications. The acetic acid solution was prepared by slowly adding 6 mL of glacial acetic acid (approximately 99% purity) into a beaker containing 144 mL of distilled water, resulting in a total volume of 150 mL. This yields approximately 4% (*v*/*v*) acetic acid. The mealworm chitosan product was transferred into a clean 200–250 mL beaker, and the prepared acetic acid solution was added slowly to the solid material. The beaker was then covered, and the mixture was stirred using a magnetic stirrer at approximately 300–400 rpm and maintained at 50 °C for 24 h to ensure complete dissolution.

#### 2.2.6. Filtration

After completing the preceding step, the final viscous product (free of large organic residues) was centrifuged. The 150 mL mixture was divided equally into four 50 mL centrifugation tubes (37.5 mL per tube). Each tube was centrifuged at 4500× *g* for 10 min. The clear, viscous supernatant was decanted into a clean 200–250 mL beaker, while the precipitate (organic residue) was discarded. Due to the solution’s high viscosity, vacuum filtration was employed to expedite the process. The supernatant was filtered under negative pressure using a Büchner funnel and qualitative filter paper. The filtered product was collected in a clean 200–250 mL beaker for subsequent steps.

#### 2.2.7. Concentration Adjustment

The chitosan solution’s concentration was standardized to 4% (*w*/*v*). To determine the initial concentration, a 10 mL aliquot of the solution was transferred to a pre-weighed Petri dish and dried in an oven at 60–80 °C for 4 h. The mass of the dried chitosan was measured, and the concentration (mg/mL) was calculated using the following formula:

Concentration % (*w*/*v*) = Mass of solute (mg)/Volume of solution (mL)

To finish, we adjusted the final volume of the solution to achieve the desired final concentration, which was 4%, via evaporation at 90 °C with supervision of the solution. For this we used the dilution equation or dilution formula that allowed us to determine the final volume and how much volume we needed to evaporate.

C1V1 = C2V2

### 2.3. Strain Sensor Fabrication

**Figure 3:**
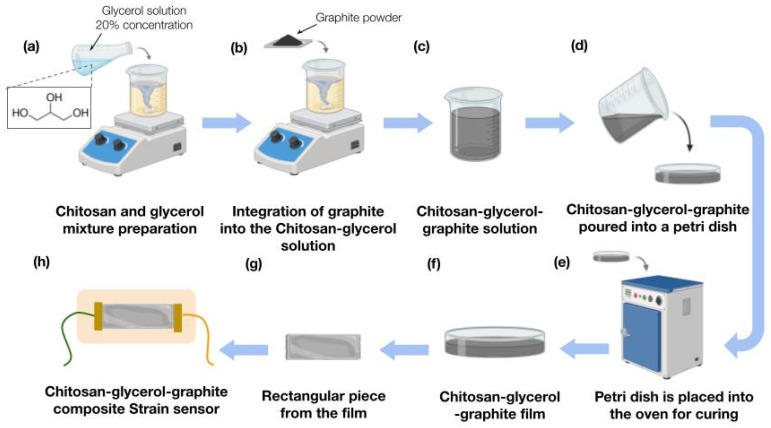
Preparation of the chitosan–glycerol–graphite composite and film formation. (**a**) A 20% glycerol solution is added to chitosan. (**b**) Graphite is gradually incorporated into the mixture under continuous stirring. (**c**) The resulting dispersion is cast onto a Petri dish. (**d**,**e**) The film is dried in an oven at 40 °C for 2 h. (**f**) The final dry composite film is shown, ready for use in sensor applications. (**g**,**h**) Show chitosan–glycerol–graphite strain sensor fabrication process. (**g**) The previously prepared film, which is manually cut into a rectangular shape with dimensions of 5 cm by 1 cm. In (**h**), wires are attached for measurements, and the strip is mounted on kinesiology tape for direct application to the patient’s skin.

For the fabrication of our sensor, we used three materials: chitosan at a 4% concentration, glycerol, and graphite powder, shown in Figure 3. The sensor design was a straight-line geometry pattern, and the dimensions were a 5 cm length and 1 cm width, with the three materials being 1 mm thick (thickness was measured using a caliper, after the film was dried). The strain sensor fabrication solution was prepared by mixing 7 mL of our previous extracted chitosan at a 4% concentration (containing approximately 0.28 g chitosan), 2.5 mL of a 10% (*v*/*v*) glycerol aqueous solution (equivalent to about 0.315 g glycerol), and 0.2 g of graphite powder. This corresponds to approximately 71.4 wt% graphite and 112.5 wt% glycerol relative to the chitosan content. The mixture was stirred at 300–400 rpm continuously for 15 min at room temperature to ensure uniform dispersion before casting and curing in oven for 2 h at 50 °C degrees and room humidity conditions (30–35%). Figure 3g,h show how the sensor was formed out of the film.

### 2.4. Strain Sensor Setup

The data acquisition system comprised an Arduino Uno microcontroller mounted on a solderless breadboard, which served as the central unit for data acquisition and system control. The microcontroller received power and facilitated serial communication via a USB interface to a host computer. A 5V DC supply from the Arduino’s 5V pin was routed to the breadboard’s power rail using a red wire, while a green wire connected the ground (GND) pin to the ground rail, establishing a common reference potential for all components. Two dedicated connection wires interfaced the breadboard with the chitosan–glycerol–graphite strain sensor. A resistor arranged in series with the sensor formed a voltage divider circuit, transducing resistance changes into quantifiable voltage signals proportional to thoracic strain. The analog output from the sensor was routed to the Arduino’s analog input pin (A0) via a shielded signal line. Voltage signals corresponding to mechanical deformation were sampled at 100 Hz, digitized by the Arduino’s 10-bit ADC, and transmitted to MATLAB R2024b for real-time visualization and post-processing.

### 2.5. Statistical Analysis

All quantitative results are presented as means ± standard deviations (SDs) unless otherwise stated. Statistical analyses were conducted using GraphPad Prism v9. For comparisons between repeated mechanical tests and sensor replicates, a one-way ANOVA was used as appropriate. A significance level of *p* < 0.05 was considered statistically significant. Due to limited sample size (*n* = 2) in human respiratory tests, only descriptive statistics (mean, SD, and coefficient of variation) were reported.

## 3. Results

### 3.1. Mechanical Characterization of the Respiratory Sensor

**Figure 4:**
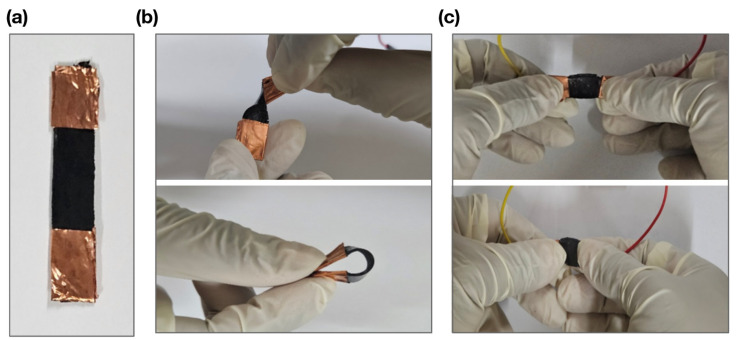
Assessment of sensor flexibility: (**a**) sensor at rest; (**b**) manual tests showing the sensor being bent and twisted; and (**c**) stretching test of the sensor from the resting position.

Preliminarily, after assembling the sensor, and before mechanical characterization, simple manual flexibility tests were carried out to assess the sensor’s ability to bend, twist, and stretch. This is illustrated in the images included in Figure 4, where the sensor is shown (Figure 4a) at rest, being bent and twisted (Figure 4b), and then stretched from the resting position (Figure 4c).

**Figure 5:**
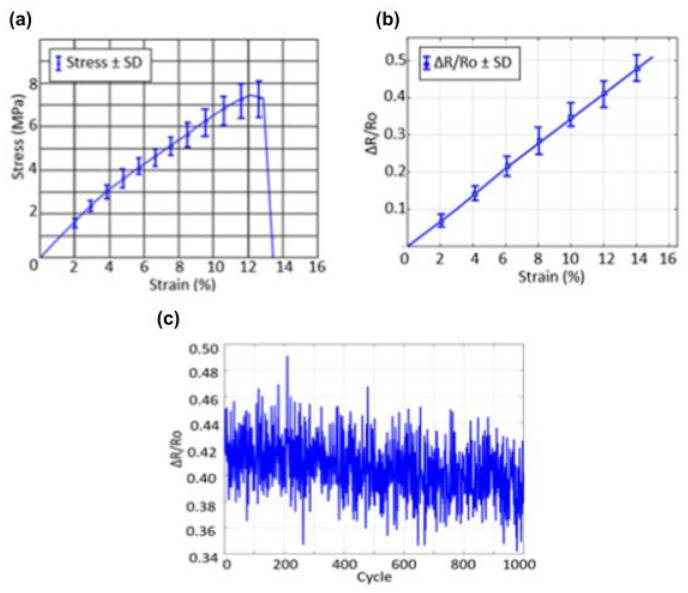
Mechanical characterization of the respiratory sensor: (**a**) stress–strain behavior of the sensor material; (**b**) relative resistance change (ΔR/R_0_) versus strain; and (**c**) cyclic durability test at 10% strain (1000 cycles). Error bars represent standard deviation (SD) across *n* = 3 sensors. Data are expressed as mean ± SD.

**Figure 6:**
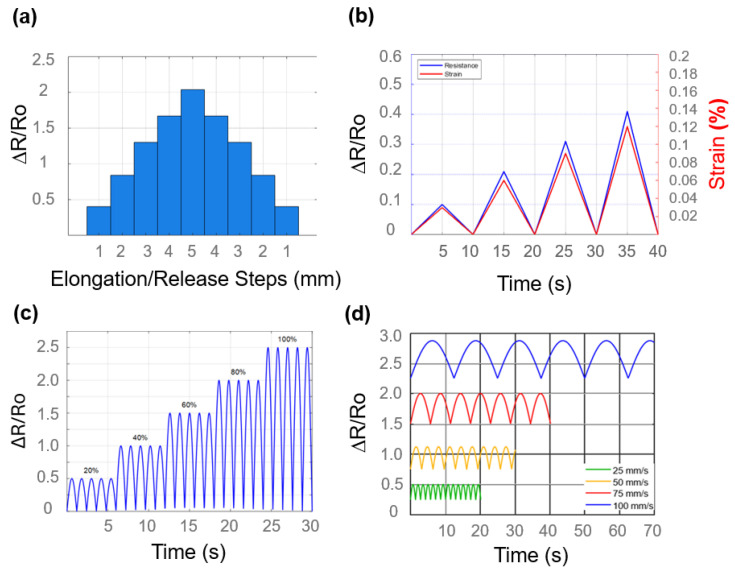
(**a**) Relative resistance change (ΔR/R_0_) across sequential elongation and release steps. (**b**) Comparison of relative resistance change and corresponding strain during cyclic loading. (**c**) Resistance changes during incremental elongation and relaxation (**d**) Real-time resistance response (ΔR/R_0_) of the sensor at varying strain rates.

After testing the flexibility manually, mechanical characterization of the sensor was carried out. The presented data demonstrate comprehensive mechanical characterization of a stretchable respiratory sensor, revealing critical performance parameters essential for respiratory monitoring applications. The experiment results (Figure 5a–c) show acceptable behavior in both mechanical stress–strain response and electrical resistance change. The mechanical properties of the synthesized sensor were evaluated utilizing uniaxial tensile testing, with the resultant stress–strain curve delineated in Figure 6a. The curve reveals three distinct phases: an initial linear elastic region, a subsequent nonlinear plastic deformation phase, and a precipitous decline indicative of fracture. During the elastic phase, the stress exhibits a linear increase in correlation with strain up to approximately 10%, implying that the sensor preserves its structural integrity under typical biomechanical strains encountered during human locomotion or respiration. Beyond this threshold, the material underwent plastic deformation prior to rapid failure, signifying ductile behavior with localized initiation of fracture. The Young’s modulus (E), derived from the slope of the linear elastic region (spanning 2% to 10% strain), was determined to be approximately.

$$E=\frac{\Delta \sigma }{\Delta \varepsilon }$$

The average Young’s modulus was 62.5 ± 3.2 MPa (*n* = 3). A one-way ANOVA confirmed no significant difference across samples (*p* > 0.05), indicating consistency in mechanical behavior.

This intermediate stiffness substantiates that the composite is both mechanically compliant and resilient, rendering it highly appropriate for small-range movements, such as rib movements during the respiratory cycle, that necessitate frequent elongation and relaxation without compromising signal stability. The integration of glycerol as a plasticizer augments flexibility and elasticity, whereas graphite contributes to electrical conductivity and mechanical fortification. Chitosan, a biodegradable biopolymer, offers a flexible and biocompatible matrix, thereby enhancing environmental sustainability and biocompatibility. This amalgamation of materials yields a composite that is sufficiently pliable to adapt to dynamic skin movements, robust enough to endure operational strains, electrically active due to the incorporation of graphite, and environmentally sustainable, with prospective applications in disposable respiratory monitoring patches and eco-friendly wearable electronics.

The sensor’s response in Figure 5b was linear up to 15% strain, and the overall change in resistance was roughly 52%. This behavior indicates electrical performance. The performance remains consistent and is easy to predict even when the material is bent. We found the gauge factor (GF), which shows how sensitive the resistive response is to strain, to be

$$GF=\frac{\Delta R/R}{\varepsilon }$$

The gauge factor (GF) across three samples was 3.47 ± 0.21 (mean ± SD, *n* = 3). A one-way ANOVA showed no significant variation between replicates (*p* = 0.39).

To quantify sensor sensitivity, we calculated the gauge factor (GF) across three different samples and averaged the results. The GF was 3.47 ± 0.21, aligning with or exceeding values reported for biopolymer-based resistive strain sensors, such as CNT hybrids (GF ≈ 2.1) [34]. This highlights the chitosan–graphite sensor competitiveness and suitability for precision bio-motion monitoring.

To demonstrate the reproducibility and reliability of the fabricated sensor, standard deviation error bars were incorporated into both the mechanical (stress–strain) and electrical (resistance–strain) performance plots, as shown in Figure 5 and Figure 6. Each data point represents the average of three independent measurements, and the error bars (±SD) indicate the variability across repeated tests. The relatively small error margins observed in both plots confirm the consistency of sensor behavior under repeated mechanical loading, highlighting its structural integrity and stable sensing performance. The calculated Young’s modulus is approximately 62.5 ± 3.2 MPa, which indicates a moderate stiffness suitable for wearable applications. This modulus places the sensor in a favorable position compared to conventional soft polymer-based strain sensors. For example, PDMS (polydimethylsiloxane)-based sensors typically exhibit a Young’s modulus in the range of 0.36–2.5 MPa [35,36], offering excellent flexibility but limited mechanical strength. Similarly, Ecoflex-based elastomer sensors exhibit Young’s moduli of 125–150 kPa [37]. While these materials offer ultra-softness for skin-like conformity, their mechanical robustness is often insufficient for structural applications requiring both sensitivity and durability. Figure 5 shows the mechanical characterization of the respiratory sensor, depicting (a) the stress–strain behavior of the sensor material and (b) the relative resistance change (ΔR/R_0_) versus strain. In (a) and (b), the experimental setup durability is shown. The higher modulus of the proposed chitosan–graphite-based sensor suggests improved mechanical reinforcement and load-bearing capability while maintaining acceptable flexibility, making it suitable for repeated wear and strain without structural fatigue.

Additionally, the resistance–strain plot (Figure 5b) shows a highly linear and reproducible piezoresistive response, with a consistent relative resistance change (ΔR/R_0_) across the tested strain range (0–15%). The inclusion of error bars further confirms that the electrical performance of the sensor remains stable and repeatable under mechanical deformation. This reproducibility, combined with its favorable mechanical modulus, demonstrates the sensor’s potential for reliable operation in wearable strain sensing applications, including respiratory monitoring, joint movement detection, and posture analysis. The observed mechanical characteristics align with the intended functionality of the sensor, particularly its reliable mechanical deformation under low-stress conditions typical of monitoring respiration or joint movements. These results, combined with the good Young’s modulus (~±3.2 MPa) observed by mechanical testing, suggest that the chitosan–glycerol–graphite composite is a good choice for bioelectronic strain sensors that are safe for skin, good for the environment, and require little electricity.

Figure 5c shows the durability performance of the strain sensor over 1000 cycles at 10% strain. The sensor retained 94.5% ± 1.8% of its original ΔR/R_0_ signal, with only minimal degradation observed over time. This result confirms the mechanical robustness and electrical repeatability of the sensor under cyclic deformation conditions relevant to pathological breathing.

### 3.2. Strain Sensor Analysis

Figure 6 clarifies the normalized resistance fluctuation (ΔR/R_0_) of the sensor over a range of incremental strain intensities from 2% to 10%. Each apex denotes a loading–unloading cycle, with the amplitude of ΔR/R_0_ demonstrating amplification in response to the level of strain. The trend validates the sensor’s elevated strain sensitivity and commendable cyclic repeatability. The signal exhibits uniform waveform forms over cycles, signifying stability under dynamic stress.

Figure 6b illustrates the sensor’s resistance response under continuous strain while adjusting the strain rates from 25 to 100 mm/s. The oscillations in resistance are clearly discernible at each strain rate. As the strain rate increases, the amplitude of the resistance fluctuations intensifies, signifying a sensing response that is contingent upon the rate. This phenomenon may be attributed to the viscoelastic characteristics of the sensing material, or the frictional interactions present within the composite structure.

The uniformity of waveform patterns at varying rates indicates rapid response and recovery attributes. The histogram depicts ΔR/R_0_ in relation to incremental elongation (1–5 mm) and the associated release stages. The ΔR/R_0_ data exhibits a linear increase during elongation and a symmetrical return during release, indicating favorable linearity and reversibility of the sensor. This graph confirms the sensor’s dependable bidirectional capability and its proficiency in precise strain measurements across numerous cycles.

This dual-axis graph compares ΔR/R_0_ (left, blue) with the actual strain % (right, red) over time. The two graphs demonstrate a strong correlation throughout cycles, indicating a significant relationship between the electrical signal and mechanical deformation. The almost simultaneous action illustrates the sensor’s proficiency in accurate strain monitoring for real-time applications. Figure 6d presents the resistance response (ΔR/R_0_) of the sensor under different strain rates (25, 50, 75, and 100 mm/s) applied sequentially. The data demonstrates that the amplitude of the resistance change increases with strain rate, with distinct waveforms corresponding to each rate. This behavior highlights the sensor’s sensitivity to strain velocity, likely due to the viscoelastic characteristics of the chitosan–glycerol matrix and dynamic interfacial friction within the composite. The clear separation between the signal levels also confirms the sensor’s ability to differentiate motion speeds, making it suitable for detecting not just deformation but also motion dynamics.

### 3.3. Respiratory Pattern Characterization

**Figure 7:**
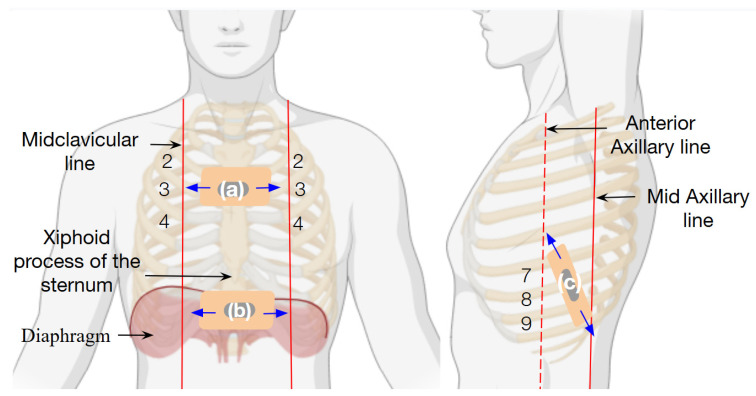
Sensor placement on the thoracic cage: (**a**) the strain sensor was placed between the 2nd and 4th intercostal spaces (showed in black numbers in the figure) over the sternum to measure anterior chest movement; (**b**) the second area was below the xiphoid process to detect diaphragm motion; and (**c**) finally, it was placed between the 7th and 9th intercostal spaces to capture lower rib expansion. Blue arrows indicate the direction of strain. During quiet breathing, anterior expansion ranges from 1 to 2 cm, and anterolateral from 2 to 5 cm.

For respiratory characterization, the strain sensor performance was tested in different thoracic areas in healthy volunteers who mimicked respiratory patterns following verbal instructions. All respiratory patterns simulated in this study and sensor placement were designed and conducted under the guidance of a healthcare professional (licensed kinesiologist with clinical experience in pulmonary rehabilitation and intensive care). The patterns were selected to represent physiologically relevant breathing conditions for reliability purposes. As shown in Figure 7, the chitosan–glycerol–graphite strain sensor was placed on three different areas of the thoracic cage to evaluate its efficacy and demonstrate its functionality.

First, the sensor was positioned in area (a) (Figure 7a), located between the midclavicular lines at the level of the second, third, and fourth intercostal spaces, covering the sternum region. The second placement was in area (b) (Figure 7b), situated at the level of the diaphragm’s excursion, just below the xiphoid process of the sternum. Finally, in area (c) (Figure 7c), the sensor was placed along the lower ribs between the anterior and mid-axillary lines, approximately at the seventh, eighth, and ninth intercostal spaces, positioned perpendicularly to the direction of the ribs.

**Figure 8:**
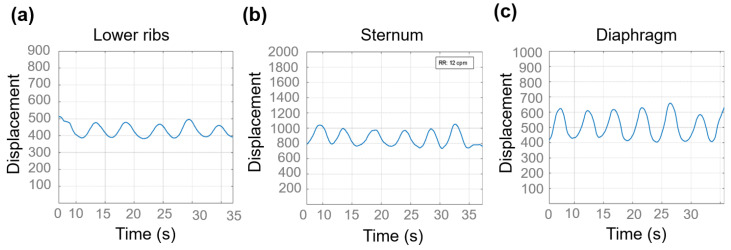
Relative resistance change (ΔR/R_0_) defined as displacement of the thoracic cage in (**a**) the lower rib area, (**b**) sternum, and (**c**) diaphragm, during respiratory cycles of 30 s.

The sensor’s activity is reflected as changes in resistance over time during respiratory cycles and is illustrated in Figure 8a–c. These resistance changes correspond to thoracic movements occurring during each respiratory phase, which include inhalation and exhalation, demonstrating the sensor’s ability to sense thoracic expansion and relaxation throughout a normal respiratory cycle. The plot in this study was intentionally designed to display the respiratory pattern in real time, similar to a clinical monitor. The 30 s window was selected solely for visual presentation purposes.

**Figure 9:**
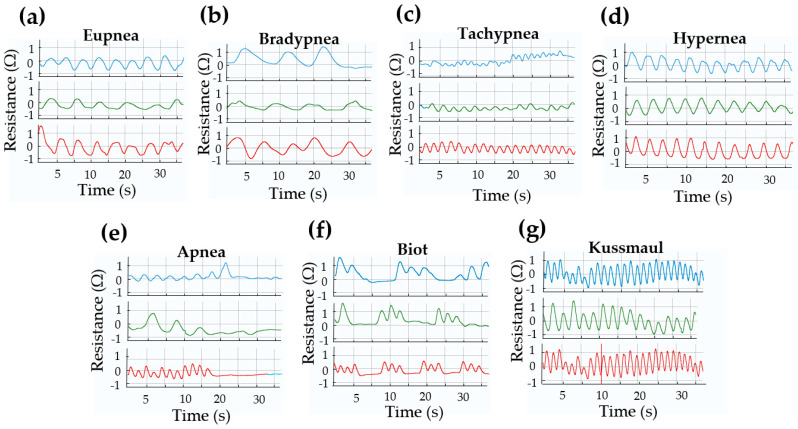
Respiratory patterns recorded using a strain sensor placed at three thoracic locations: lower ribs (blue), sternum (green), and diaphragm (red). The plots show distinct breathing patterns: (**a**) eupnea, (**b**) bradypnea, (**c**) tachypnea, (**d**) hyperpnea, (**e**) apnea, (**f**) Biot’s respiration, and (**g**) Kussmaul breathing.

To further evaluate sensor functionality, distinct respiratory patterns were measured across the three thoracic locations, as presented in Figure 9. These plots represent the relative resistance change (ΔR/R_0_) over time. The zero value on each vertical axis corresponds to the baseline resistance at rest (R_0_), and the curves show deviations from this baseline during thoracic expansion and contraction. In Figure 9a, the sensor records a normal breathing pattern (eupnea), which is defined as a regular respiratory cycle with a rate between 12 and 30 breaths per minute (bpm) in adults. Figure 9b illustrates bradypnea, a slower breathing pattern with a rate below 12 bpm, while Figure 8c shows tachypnea, characterized by a respiratory rate exceeding 30 bpm. Similarly to tachypnea, hyperpnea (Figure 9d) is marked by an increased amplitude (i.e., deeper breaths), often occurring alongside tachypnea.

The sensor captures the characteristic thoracic motion associated with each pattern across different anatomical regions.

The absence of respiratory activity is represented in apnea (Figure 9e), where the person temporarily stops breathing. In such cases, the sensor output approaches zero, indicating minimal or no thoracic movement. Clinically, apnea episodes can last about 20 s and may result from various pharmacological or pathological causes, including respiratory arrest. The plot shows 30 s, but the relevance is the representation of the interruption of the signal. Other pathological respiratory patterns are also identifiable: Biot’s pattern (Figure 9g), associated with damage to the medulla oblongata or pons, increased intracranial pressure, or opioid overdose, presents an abnormal breathing pattern, where brief periods of about three respiratory cycles with increased frequency and amplitude are identified, followed by a period of apnea. Kussmaul respiration is distinguished by both increased amplitude and frequency (tachypnea) and is commonly a compensatory response to metabolic acidosis. This condition may result from diabetic ketoacidosis or renal failure (severe metabolic acidosis), where the body attempts to expel excess CO_2_ by breathing faster and more deeply. The sensor successfully senses physiological respiratory signals by capturing both frequency and amplitude under various breathing situations, giving us characteristic waves morphologies. The results demonstrate its capability to monitor physiological respiratory signals in real time. These findings suggest that the system holds potential for continuous respiratory signal acquisition in both clinical and home-based settings.

## 4. Discussion

Numerous wearable respiratory sensors have been proposed over the past decade, using a variety of mechanisms such as piezoresistive fabrics, strain-sensitive elastomers, fiber-optic belts, and capacitive stretch sensors. For example, studies [38] De Luca et al. proposed a fully textile-based respiratory belt with high repeatability and good agreement with spirometry measurements, while others [39] demonstrated a soft wireless sensor for continuous respiratory monitoring. More recent approaches [40,41], employed multifunctional materials and advanced integration for clinical-grade applications. Compared to these systems, our approach offers a low-cost, biocompatible alternative that uses naturally derived chitosan in combination with glycerol and graphite. Although the gauge factor and long-term stability may still require optimization for clinical deployment, our composite material showed the capacity to sense breathing patterns (including apnea and Kussmaul respiration) in real time. Furthermore, the fabrication method is simple, reproducible, and does not require specialized facilities, which opens possibilities for use in low-resource settings.

In this work, it was possible to create and obtain a respiratory sensor based on three ingredients, with a 4% chitosan concentration being the most prominent. Its viscoelastic properties, combined with the plasticizing properties of glycerol, enabled the creation of a flexible material that does not break when bent while also being stable and strong enough to withstand elastic changes under mechanical stress (stretching). The sensor’s performance was further enhanced by adding the necessary amount of graphite to provide electrical properties without altering the mechanical properties already provided by the chitosan and glycerol. This strain sensor demonstrated the ability to detect subtle movements, such as bucket handle and pump handle movements of the ribs during a normal respiratory cycle, as well as more pronounced movements in abnormal situations, like tachypnea or hyperpnea. The sensor was able to detect variations in both movement amplitude and frequency based on resistance changes as it stretched during chest expansion. This allowed for the identification of differences in respiratory effort, such as increased amplitude during deep inhalation and decreases during exhalation. The sensor successfully captured and distinguished characteristic respiratory patterns, including those typically observed in clinical respiratory assessments. Additionally, the strain sensor showed positive behavior and potential regarding mechanical robustness and durability over time. Also, the performance was demonstrated when it was tested in three different strategic areas of the thorax, all key in respiratory biomechanics: the sternum area, where anterior movement predominates, to detect anterior chest expansion; the diaphragmatic area, where the sensor was placed at the diaphragm’s excursion point to directly sense its movement (this muscle being the most important in respiratory mechanics); and the area of the lower ribs, around the 7th to 9th intercostal spaces, where greater anterolateral rib movement occurs, resulting in greater lateral thoracic expansion. The measurements obtained were consistent and coherent with each other, with clear and identifiable respiratory patterns in each case, especially when changes in amplitude and frequency were clear and stable.

### Futures Directions

Future improvements could focus on enhancing the sensor composites to achieve a better component ratio and create a more elastic material. This would allow its use in other body regions with a greater range of motion than the thorax, which requires more durable and elastic materials. Even for thoracic kinetics, more elastic material is necessary to ensure better readings and better wave morphology in clinical situations where respiratory amplitudes and frequencies are irregular, when patients are in respiratory distress, like the Kussmaul pattern. Additionally, a non-operator-dependent calibration method should be developed, along with improvements to sensor adhesion, to make its installation and fixation to the patient’s skin less cumbersome.

It is essential to consider environmental effects—specifically temperature and humidity—on the strain sensor for better performance. Human skin temperature changes and storage conditions can influence sensor performance. Chitosan can absorb atmospheric moisture, while prolonged exposure to high humidity conditions can cause swelling, and may result in performance drift or degradation over time. Regarding temperature changes, chitosan-carbon composite sensors, for example, show a negative temperature coefficient, meaning that ΔR/R0 decreased when the temperature increased. The NTC effect is caused by the fact that an increase in temperature promotes the migration of charge carriers, resulting in a decrease in resistance. The temperature response is generally monotonic and linear, between 25 and 50 °C, but extreme temperatures may accelerate degradation or induce irreversible changes in the polymer matrix. Compared with the temperature of the human body (37 °C and around 39–40 °C during fever), drastic resistance changes are not expected within this physiological range. However, humidity, especially from sweat, remains a more significant factor affecting performance. As a mitigation strategy, encapsulation techniques are viable. Applying a flexible, hydrophobic polymer coating encapsulation layer (e.g., HDF PA) can effectively shield the sensor from environmental moisture and temperature fluctuations while preserving flexibility and skin conformity. Recent studies have shown that such encapsulation reduces weight and resistance changes under high humidity, ensuring long-term stability for wearable applications [25].

Finally, this type of measurement has potential for continued use in respiratory monitoring. Features like a respiratory rate counter or the ability to estimate other clinically important respiratory parameters, such as tidal volume, could be added. Tidal volume is typically obtained invasively through pulmonary volume measurements during mechanical ventilation or via specific pulmonary function tests like spirometry, which require a trained professional to conduct, guide, and interpret the test. It can also be estimated through mathematical formulas, such as Broca’s or ideal weight formulas, which approximate a theoretical tidal volume but not the patient’s actual tidal volume. To achieve this, a clinical trial or a medical interventional study might be conducted to study the performance of the sensor in a real clinical situation.

## 5. Conclusions

This research presents the development and evaluation of a flexible, stretchable, and biodegradable respiratory sensor based on a chitosan–graphite-glycerol composite extracted from mealworm (*Tenebrio molitor* larvae). The sensor leverages chitosan’s biocompatibility, hygroscopicity, and film-forming capabilities, combined with the enhanced electrical conductivity and sensitivity provided by graphite. Sustainable extraction of chitosan from mealworm exoskeletons, coupled with straightforward film fabrication techniques, highlights the sensor’s promise for future applications.

The biodegradable and biocompatible properties of the chitosan–graphite composite ensure skin safety and environmental benefits, minimizing electronic waste. Its lightweight, flexible construction enhances wearability and overcomes the limitations of traditional bulky respiratory monitoring equipment, thus improving patient mobility and comfort. However, further development is needed to ensure operational stability under extended, real-world conditions. Environmental factors such as humidity, temperature fluctuations, and mechanical stress may affect long-term electrical performance. Therefore, environmental stability tests and accelerated aging experiments are necessary to evaluate performance degradation, moisture absorption, and mechanical fatigue.

In summary, the stretchable chitosan–graphite sensor provides an affordable and sustainable option for wearable respiratory physiological signal monitoring. Future research should focus on optimizing material composition and sensor design, integrating wireless data transmission, and validating performance in real-world clinical conditions for future clinical use.

## Data Availability

The original contributions presented in the study are included in the article, further inquiries can be directed to the corresponding authors.
